# Revisiting multiple erroneous genetic testing results and clinical misinterpretations in a patient with Li-Fraumeni syndrome: lessons for translational medicine

**DOI:** 10.1186/s13053-020-00157-8

**Published:** 2021-01-06

**Authors:** Tatiana N. Sokolova, Valeriy V. Breder, Irina S. Shumskaya, Evgeny N. Suspitsin, Svetlana N. Aleksakhina, Grigoriy A. Yanus, Vladislav I. Tiurin, Alexandr O. Ivantsov, Barbara Vona, Grigoriy A. Raskin, Sergey V. Gamajunov, Evgeny N. Imyanitov

**Affiliations:** 1 N.N. Petrov Institute of Oncology, Pesochniy, Saint-Petersburg, 197758 Russia; 2 N.N. Blokhin Russian Cancer Research Center, Moscow, 115478 Russia; 3 Regional Cancer Hospital, Nizhniy Novgorod, 603093 Russia; 4 St.-Petersburg Pediatric Medical University, Saint Petersburg, 194100 Russia; 5 Tübingen Hearing Research Centre, Eberhard Karls University Tübingen, 72076 Tübingen, Germany; 6 A.M. Granov Russian Scientific Center of Radiology and Surgical Technologies, Saint Petersburg, 197758 Russia

**Keywords:** Li-Fraumeni syndrome, Breast cancer, Lung cancer, *TP53*

## Abstract

**Background:**

Many cancer patients undergo sophisticated laboratory testing, which requires proper interpretation and interaction between different specialists.

**Case presentation:**

We describe a patient with an extensive family history of cancer, who was diagnosed with bilateral breast cancer and two lung cancer lumps by the age of 40 years. She submitted a lung cancer specimen to a genetic profiling service, which reported the presence of the *EGFR* mutation (a combination of G719S and L833V substitutions) and the *TP53 с.322_327del* (p.G108_F109del) mutation in the tumor tissue. Possible therapeutic options were discussed at a medical conference, where one of the discussants raised a concern that the identified *TP53* mutation may not necessarily be somatic, but reflect the germ-line status of the gene. Review of clinical records and follow-up dialog with the patient revealed, that she previously provided her blood for DNA analysis in two laboratories. The first laboratory utilized a custom NGS assay and did not detect the *TP53* mutation, instead pointed to a potential pathogenic significance of the *MSH6 c.2633 T > C* (p.V878A) allele. The second laboratory revealed the *TP53 с.322_327del* (p.G108_F109del) allele but stated in the written report that it has an unknown pathogenic significance. To resolve the possible uncertainty regarding the role of the *TP53 с.322_327del* (p.G108_F109del) variant, we suggested that the patient invite her second cousin for genetic testing, as she was affected by neuroblastoma at the age of 3 years. This analysis revealed the presence of the same *TP53* variant.

**Conclusion:**

We provide point-by-point discussion, reviewing multiple laboratory mistakes and clinical misinterpretations occurred with this patient. This case report exemplifies the need to involve rigorous clinical expertise in the daily practice of medical laboratory facilities.

## Background

Management of malignant disease is an enormously complex process involving surgeons, medical oncologists, radiologists, pathologists etc. In addition to conventional analyses, many cancer patients undergo sophisticated mutation tests, which are usually done in pathology departments or specialized genetic facilities. It is unavoidable, that every expert involved in the treatment of a given patient focuses only on particular aspects of the cancer disease, hence the importance of multidisciplinary teams in the organization of optimal cancer treatment has been repeatedly emphasized [1]. Here we present an intriguing story of a cancer patient who was subjected to a series of state-of-the-art tests and received counseling in a number of well-qualified clinical centers, but eventually received a diagnosis of Li-Fraumeni syndrome solely by chance. We believe that a point-by-point analysis of the multiple medical errors occurring during the examination of this patient emphasizes the existing challenges and limitations in translational medicine.

## Case presentation

Patient R. was presented in a cancer meeting held in Moscow, in March 2020, in a clinical discussion of the results of the Foundation Medicine genomic tumor profiling test. One of the authors of this report, who was present at this meeting, raised a concern regarding the interpretation of the test. The Foundation Medicine report indicated the presence of the *TP53 с.322_327del* (p.G108_F109del) mutation in the analyzed tumor. The discussant argued that the young age of the patient and presence of primary multiple tumors are compatible with a Li-Fraumeni syndrome diagnosis [2–5], so this *TP53 с.322_327del* (p.G108_F109del) mutation may not necessarily be somatic. There were other apparent inconsistencies, so the two primary physicians, who were in charge of this patient, expressed a willingness to cooperate and invited the patient to continue her medical and genetic examination. The patient immediately responded to the invitation, provided her clinical records and biological samples for investigation and expressed enthusiasm regarding the dissemination of the gained information within the medical community.

**Fig. 1:**
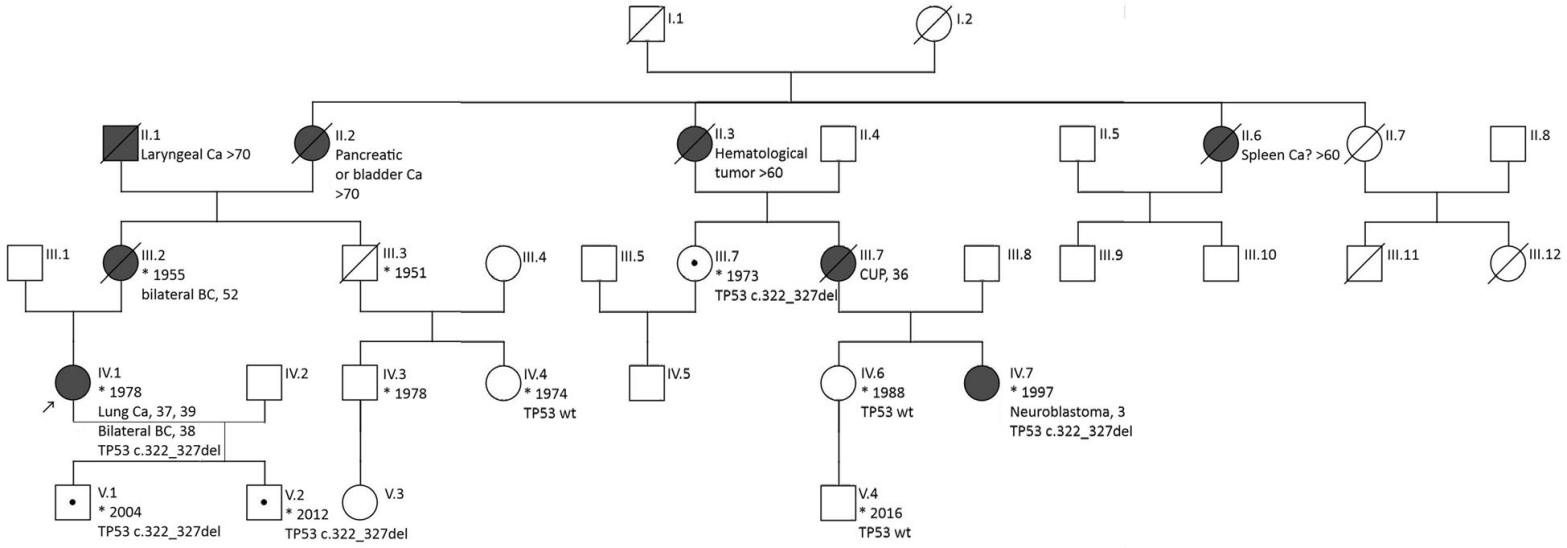
Pedigree of the patient described in this study

Patient R is a female, born in the year 1978, and belongs to a pedigree with multiple instances of cancer (Fig. 1). Her mother had bilateral breast cancer (BC) and died at the age of 52 years. There were cancers in grandmother of the patient, and two of the sisters of this grandmother suffered from malignancies as well. There were instances of the young-onset cancer of unknown primary site and the childhood neuroblastoma in the cousins of the patient.

The patient was diagnosed with her first tumor in March 2016. Despite being a non-smoker, she developed lung adenocarcinoma in the left upper lobe and underwent lobectomy. The tumor had apparently favorable staging, T1aN0M0, and did not contain *EGFR* mutation or *ALK* rearrangement according to laboratory reports. The patient subsequently detected a lump in her right breast during self-examination, and underwent surgery for breast cancer in April 2017. The morphological examination revealed breast cancer in situ, ER/PR-positive, HER2+++. Also, during the spring of 2017, the patient revealed a lump in the contralateral breast. The biopsy identified triple-negative BC, and the woman was subjected to a neoadjuvant therapy (doxorubicin and paclitaxel) from May 2017 to November 2017. The surgery was performed in November 2017. The excised tumor was classified as T2 N0M0; in contrast to the biopsy material, it demonstrated immunohistochemical (IHC) staining for ER, while the status of PR and HER2 remained negative. The amount of the biopsied tissue was insufficient to repeat IHC, so it was unclear whether there was a true change of ER expression during neoadjuvant therapy, or if some technical variations of IHC assays contributed to this disagreement.

The primary physician was concerned by the young age of the patient, the emergence of multiple tumors and the extensive family history of cancer, and forwarded this woman to genetic analysis. According to the report from the regional genetic laboratory, germline DNA analysis was performed by next generation sequencing (NGS) using a custom target enrichment panel. This panel contained over 50 genes with known involvement in hereditary cancer predisposition, including *TP53*. This NGS analysis did not identify mutations in *BRCA1/2* or *TP53* genes; however, it revealed a missense substitution *c.2633 T > C* (p.V878A) in the *MSH6* gene. The report issued by the laboratory stated that this variant affects the function of the MSH6 protein according to genetic disease databases and this substitution is pathogenic. This result was interpreted as an evidence for Lynch syndrome.

The patient was concerned by this diagnosis and repeated *MSH6* mutation testing a well-known cancer center. The presence of *MSH6 c.2633 T > C* (p.V878A) variant was confirmed. The report from the genetic laboratory stated that this variant occurs in the population at a frequency of 0.004 and is of unknown clinical significance. Despite this interpretation this report recommended to analyze the presence of this variant in the relatives of the patient.

The patient went to a third genetic service, this time to a unit located in a distinguished clinical genetic center. She received a counseling report ensuring that the previously identified *MSH6 c.2633 T > C* (p.V878A) variant was a gene polymorphism, which was unlikely to have a clinical significance. She was also offered a test for the *TP53* gene, given that the pattern of the tumors within her family was compatible with the diagnosis of the Li-Fraumeni syndrome. The germline DNA test also identified the *с.322_327del* (p.G108_F109del) allele. However, the accompanying report stated that this *TP53* variant was of unknown clinical significance, and no further recommendations were provided.

Meanwhile, a routine CT examination in October 2018 revealed a lump in the lower lobe of the left lung. This lump increased in size after 6 weeks of follow-up, and the lobectomy was performed in December 2018. The histological examination confirmed the presence of lung adenocarcinoma. This finding was interpreted as a metastatic spread of previously excised lung cancer (LC). The patient went on observation.

In May 2019 new lumps in the lung were detected, and the tumor tissues obtained from this patient were subjected to further analysis. The first BC and the recently excised LC lump were subjected to IHC testing for MLH1, MSH2, PMS2 and MSH6 commercial diagnostic antibodies. The analysis revealed the loss of MSH6 expression in both tumors. The pathological report stated that the tumor tissue had microsatellite instability and recommended genetic testing for Lynch syndrome. The LC tissue obtained at surgery in December 2018 was also subjected to *EGFR* mutation analysis and IHC testing for *ALK* and *ROS1* activation; however, no actionable mutations were identified. Nonetheless, PD-L1 IHC with the 22C3 antibody revealed staining in 65% of tumor cells. Based on the results of these analyses, the patient was administered single-agent pembrolizumab starting from August 2019. This therapy did not stop enlargement of tumor lumps, therefore pemetrexed and cisplatin were added to pembrolizumab in January 2020. This combination of the inhibitor of immune checkpoints and chemotherapy led to an evident reduction of the size of metastatic lesions.

The same LC sample was forwarded to the Foundation Medicine for genomic profiling. Surprisingly, the testing revealed *EGFR* mutation (a combination of G719S and L833V substitutions); this was in controversy with a previous *EGFR* testing report, which included the G719S substitution but claimed a normal status of the codon 719. The genomic profiling also identified the *TP53 с.322_327del* (p.G108_F109del) mutation as well as some other genetic alterations (*CCNE1* amplification, *CDC73* inversion exon 3, *RAD21* amplification). The tumor was found to be microsatellite stable. The 18-page report contained a half-page overview on *TP53* gene biology and its potential predictive value. It was properly mentioned in the last paragraph of this overview that germline *TP53* pathogenic variants “are associated with the very rare disorder Li-Fraumeni syndrome and the early onset of many cancers”. The last sentence in the *TP53* finding summary stated that “in the appropriate clinical context, germline testing of *TP53* is recommended”. However, despite the Foundation Medicine having information on the unusually young age of this lung cancer patient, a possible diagnosis of Li-Fraumeni syndrome was not mentioned in the front page of this report.

As already mentioned in the beginning of this case presentation, the patient was subsequently discussed at a medical meeting and was invited to continue genetic examination. The *TP53 с.322_327del* (p.G108_F109del) mutation is repeatedly reported in the COSMIC somatic mutation database, therefore it is likely to contribute in tumor pathogenesis. The descriptions of the Li-Fraumeni syndrome indicate that the spectrum of causative *TP53* mutations includes the entire region of the *TP53* gene [2–5]. Hence, the presence of the *TP53 с.322_327del* (p.G108_F109del) allele in the germline is itself compatible with a Li-Fraumeni syndrome diagnosis, however, no other families with this variant have been described in the literature. Furthermore, it is not entirely impossible if the loss of two amino acids at positions 108 and 109 does not critically affect the function of TP53 protein, therefore additional evidence may be needed to confirm the diagnosis. To resolve this issue, we suggested that the patient invite her second cousin, who survived neuroblastoma at the age of 3 years and obviously shared a family history of cancer, for genetic testing (Fig. 1; subject IV.7). The cousin positively responded to the invitation, and her analysis revealed the presence of the same *TP53* germline variant. Taken together, these findings led us to conclude that the diagnosis of Li-Fraumeni syndrome is a correct characteristic of the described cancer cases. We further invited other relatives for *TP53 с.322_327del* testing, and revealed a few additional carriers (Fig. 1; subjects III.6, V.1, V.2; all of them remain healthy at the time of the preparation of this report, being 47, 16 and 8 years of age, respectively). We noticed, that one of the *TP53 с.322_327del* allele carriers (III.6) achieved a decent age being cancer-free, however this is compatible with wide variations for the age at tumor onset and disease penetrance in subjects affected by Li-Fraumeni syndrome [5].

We further analyzed all four tumors obtained from this patient. Interestingly, the first lung tumor, excised in the year 2016, did not contain *EGFR* mutations, while we confirmed the presence of *EGFR* mutation (a combination of G719S and L833V substitutions) in the second lung cancer lump. These findings suggest that the second lung tumor is not a metastasis of the first lung malignancy, as was initially thought, but an independent cancer. We also analyzed the status of the remaining *TP53* allele in all four tumors. There were no instances of the deletion of the remaining *TP53* gene copy, which is well compatible with knowledge on tumors arising in Li-Fraumeni syndrome patients [2–5]. Interestingly, the first LC, but not three other tumors, contained an additional somatic mutation in *TP53* gene, *c.1010G > A* (p.R337H). It is also known as a Brazilian founder pathogenic variant [3–5], therefore we re-tested the blood DNA sample and the tumor DNAs; these experiments confirmed that this substitution is not a germline allele and that it is present only in one cancer sample. The repetition of MLH1, MSH2, PMS2 and MSH6 staining revealed normal expression status for all four proteins.

## Discussion

**Fig. 2:**
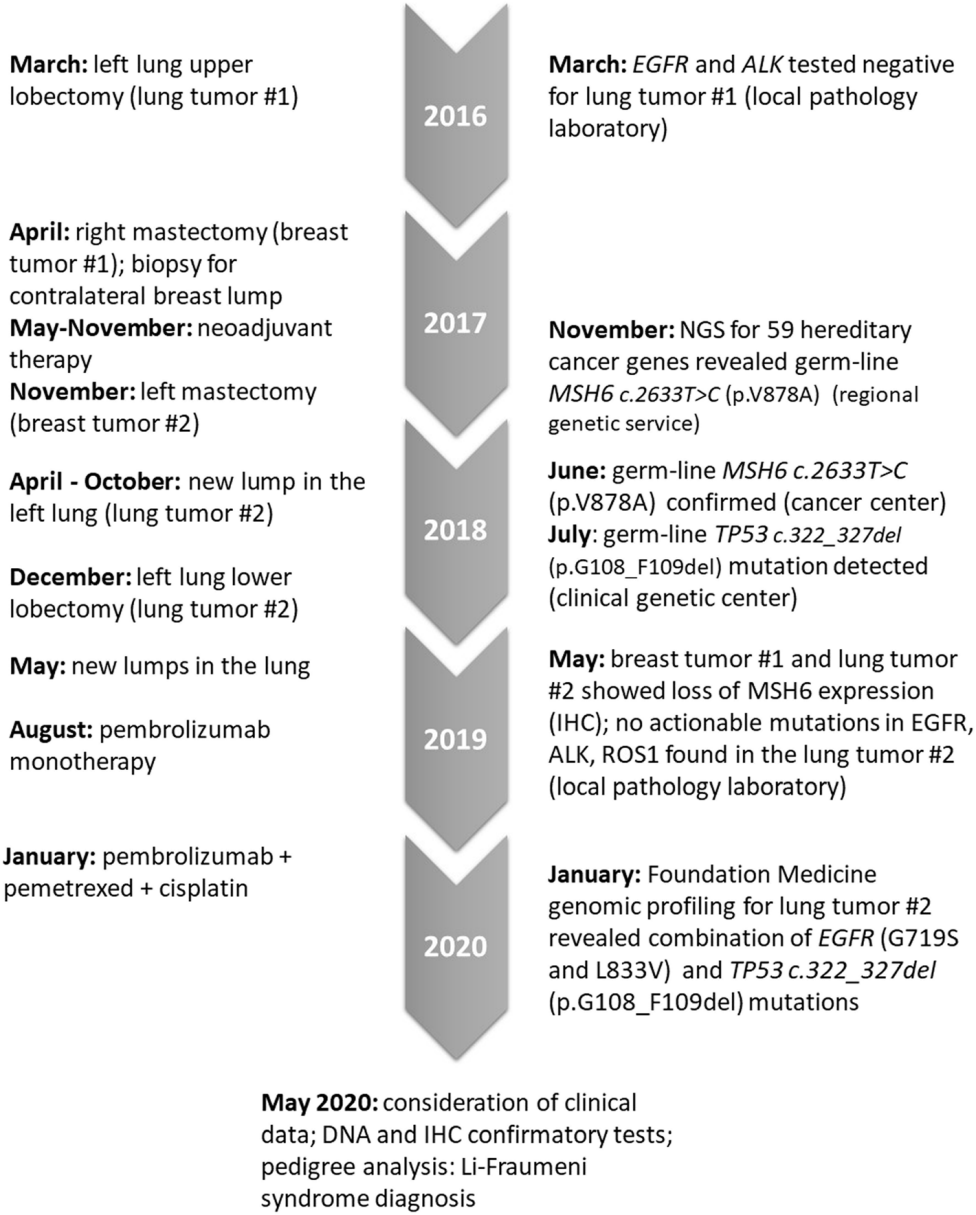
Brief history of cancer diagnoses and molecular tests assigned to patient R (Fig. 1, IV.1)

This case report presents a dramatic accumulation of frank mistakes, which occurred during the diagnostic examination of a patient with unusual cancer presentation (Fig. 2). In retrospect, some of these mistakes could have been avoided if streamlined interaction between primary physicians and laboratory specialists was in place. Most of molecular diagnostic procedures are performed on the limit of the available technologies, therefore laboratory errors may occur in some circumstances. It is unrealistic to expect that medical oncologists will maintain fluency in the rapidly evolving developments of laboratory tests, and, vice versa, one cannot claim comprehensive clinical expertise for all specialists involved in pathological or genetic analysis of biological material. We believe that a thoughtful discussion of this case may contribute to the improvement of the infrastructure for translational medicine.

The primary physician of this patient made a perfectly correct decision in the year 2017 by sending this patient to genetic testing, as the woman was diagnosed with three tumors (LC and bilateral BC) before the age of 40 years and reported an extensive cancer family history. However, NGS analysis failed to detect the existing germline *TP53* pathogenic allele. NGS is an evolving technology, which is rapidly becoming a part of clinical medicine [6]. It is extremely difficult to ensure a proper validation of existing NGS assays and services, given that by definition they deal with an indefinite spectrum of various mutations which are spread across a very high number of genes. Therefore, it is natural to expect, that NGS may be prone to artifacts, at least in some circumstances. Perhaps, if a cancer genetic specialist would have been involved in the clinical interpretation of the NGS results, he/she would have insisted to revisit the *TP53* gene analysis in this particular case, given the presence of very strong phenotypic indicators of Li-Fraumeni syndrome. The timely diagnosis of the *TP53* germline mutation could have been of some importance, as lung tumors arising in Li-Fraumeni families almost always have somatic *EGFR* mutations [7], so this diagnosis, in theory, could encourage the re-analysis of the *EGFR* gene after obtaining an initially negative *EGFR* result. Even worse, the NGS laboratory report suggested the diagnosis of Lynch syndrome based on the presence of the *MSH6* substitution with at best unproven clinical significance, which made an impact on further management of this patient. Again, if a cancer genetic expert would have been involved in the interpretation of the NGS results, he/she would have certainly noticed that the oncological diseases occurring in this pedigree are not at all compatible with a Lynch syndrome diagnosis.

The pathogenic role of the *MSH6 c.2633 T > C* (p.V878A) was later questioned by another expert involved in the management of this patient. However, the same expert recommended *MSH6 c.2633 T > C* (p.V878A) testing in the relatives of this woman without providing grounds for this advice.

The next error occurred in the year 2018, when the patient was appropriately advised to undergo *TP53* testing due to clinical suspicion of Li-Fraumeni syndrome and even received correct results indicating the presence of the *TP53 с.322_327del* (p.G108_F109del) germline variant. However, the laboratory stated that the pathogenic significance of this variant was unknown, which was probably formally true but demonstrated an obvious conflict with clinical data and with common sense. Given that a Li-Fraumeni syndrome diagnosis calls for genetic examination of the relatives of the affected proband, and the *TP53* germline testing is relevant even for children [2–5], we believe that it was a mistake not to issue a written recommendation for further clarification of the role of this allele and for consideration of familial segregation testing.

Despite knowledge about the presence of the germline *TP53 с.322_327del* (p.G108_F109del) variant, the Lynch syndrome diagnosis surfaced again after the morphological laboratory revealed the absence of MSH6 expression in tumor tissue. Instances of isolated absence of MSH6 expression in tumor tissue in combination with the retention of MLH1, MSH2 and PMS2 expression have been described in carriers of pathogenic germline *MSH6* alleles [8]; therefore, there was an apparent agreement between IHC data and the results of blood DNA *MSH6* analysis. The pathologist found these evidences sufficient to interpret the data as a microsatellite instability and to suggest the presence of Lynch syndrome. This was an unfortunate decision, given that the DNA-based microsatellite instability analysis is easily available in Russia, so it should not have been taken extraordinary efforts to transport the archival tumor sample for a proper confirmatory investigation. We believe, it was a mistake to interpret the IHC findings as an equivalent of microsatellite unstable status of the tumor. Experts working with IHC for MLH1, MSH2, PMS2 and MSH6 are advised to be aware of possible pitfalls related to this procedure [9].

The *EGFR* assay, which was performed in a local laboratory using certified commercial *EGFR* testing kit and appropriate equipment, missed a clinically relevant *EGFR* G719S mutation. This mutation was subsequently detected by the Foundation Medicine service and by a confirmatory laboratory analysis. Missed or inaccurate tumor cell dissection is a most probable cause of false-negative somatic mutation tests. The *EGFR* G719S allele is detected by allele-specific PCR, which, in theory, should be able to reveal the mutation even if only a few percent of malignant cells are present in the sample. While the analysis of common drug-sensitizing mutations in *EGFR* has been repeatedly reported in the literature, we are unaware how the operational characteristics of existing *EGFR* assays were evaluated with regard to “rare” potentially actionable mutations (G719A, G719S, G719C, L861Q etc.). Of course, the probability of human error cannot be excluded while dealing with any medical intervention. It is unclear whether the false-negative *EGFR* result would have influenced further management of the patient. Formally speaking, single-agent pembrolizumab is approved as a front-line treatment for non-small lung cancer patients, whose tumors express PD-L1 and do not contain *EGFR* drug-sensitizing mutations [10, 11]. It is self-explanatory that most patients, who were excluded from these pembrolizumab registration trials due to the results of *EGFR* test, carried exon 19 deletion or L858R substitution in the tumor tissue, while only a minority if any LC cases were represented by rare *EGFR* mutations. Obviously, the data for “rare” *EGFR* mutations are insufficient even for conclusive analysis of the degree of their actual sensitivity to tyrosine kinase inhibitors [12, 13], and it is currently impossible to decide whether the presence of a *EGFR* G719S mutation indeed favors the upfront administration of an EGFR inhibitor, or vice versa, it does not preclude the first-line administration of the immune checkpoint therapy. It is also necessary to keep in mind, that the patient experienced the emergence of four independent tumors within a short time interval, so it is not self-explanatory that the observed metastases originated from the *EGFR*-mutated LC.

The Foundation Medicine report correctly identified *EGFR* and *TP53* mutations, but in effect failed to attract attention to the need for germline testing and for consideration of Li-Fraumeni syndrome diagnosis. As result, the patient was diagnosed with Li-Fraumeni syndrome purely due to a chance, as she was introduced at a medical meeting to discuss possible options for the therapy and was presented as a case of suspected Lynch syndrome. The diagnosis of Li-Fraumeni syndrome led to subsequent genetic analysis of the relatives of this patient, so the eventual clarification of this situation is likely to have a positive medical impact.

## Conclusion

Li-Fraumeni syndrome is an orphan disease. The difficulties in the diagnosis of exceptionally rare diseases are well appreciated, and they are applicable even to highly developed countries [14]. Perhaps, many of these failures could be avoided if the medical infrastructure would better encourage interaction between different specialists. It is of concern that many pathology departments and DNA testing services appear to be disconnected from a rigorous clinical expertise and are currently viewed as more or less independent laboratory facilities. This report exemplifies that these limitations are true not only for local laboratories or particular clinical hospitals, but also for highly advanced world leaders in the field of cancer medicine. We believe that it is indeed very important to consider laboratory findings in a proper clinical context. The instances of discrepancies between genetic and phenotyping data should be subjected to a thorough and responsible investigation to avoid erroneous diagnoses.

## Data Availability

The datasets used and/or analyzed during the current study are available from the corresponding author on reasonable request.

## Abbreviations

BC: Breast cancer
CT: Computed tomography
CUP: Cancer of unknown primary site
IHC: Immunohistochemistry
LC: Lung cancer
NGS: Next generation sequencing
PCR: Polymerase chain reaction

## References

[1] Selby P, Popescu R, Lawler M, Butcher H, Costa A (2019). The value and future developments of multidisciplinary team Cancer care. Am Soc Clin Oncol Educ Book.

[2] Bougeard G, Renaux-Petel M, Flaman JM, Charbonnier C, Fermey P, Belotti M (2015). Revisiting Li-Fraumeni syndrome from TP53 mutation carriers. J Clin Oncol.

[3] Guha T, Malkin D (2017). Inherited TP53 mutations and the Li-Fraumeni syndrome. Cold Spring Harb Perspect Med.

[4] Valdez JM, Nichols KE, Kesserwan C (2017). Li-Fraumeni syndrome: a paradigm for the understanding of hereditary cancer predisposition. Br J Haematol.

[5] Amadou A, Achatz MIW, Hainaut P (2018). Revisiting tumor patterns and penetrance in germline TP53 mutation carriers: temporal phases of Li-Fraumeni syndrome. Curr Opin Oncol.

[6] Yohe S, Thyagarajan B (2017). Review of clinical next-generation sequencing. Arch Pathol Lab Med.

[7] Mezquita L, Jové M, Nadal E, Kfoury M, Morán T, Ricordel C (2020). High prevalence of somatic oncogenic driver alterations in patients with NSCLC and Li-Fraumeni syndrome. J Thorac Oncol.

[8] Liu Y, Wang M, Chen Q, Zheng Q, Li G, Cheng Q, Liu S (2019). A novel heterozygous large deletion of MSH6 gene in a Chinese family with lynch syndrome. Gene.

[9] Markow M, Chen W, Frankel WL (2017). Immunohistochemical pitfalls: common mistakes in the evaluation of lynch syndrome. Surg Pathol Clin.

[10] Reck M, Rodríguez-Abreu D, Robinson AG, Hui R, Csőszi T, Fülöp A (2016). Pembrolizumab versus chemotherapy for PD-L1-positive non-small-cell lung Cancer. N Engl J Med.

[11] Mok TSK, Wu YL, Kudaba I, Kowalski DM, Cho BC, Turna HZ (2019). Pembrolizumab versus chemotherapy for previously untreated, PD-L1-expressing, locally advanced or metastatic non-small-cell lung cancer (KEYNOTE-042): a randomised, open-label, controlled, phase 3 trial. Lancet.

[12] Gristina V, Malapelle U, Galvano A, Pisapia P, Pepe F, Rolfo C (2020). The significance of epidermal growth factor receptor uncommon mutations in non-small cell lung cancer: a systematic review and critical appraisal. Cancer Treat Rev.

[13] Harrison PT, Vyse S, Huang PH (2020). Rare epidermal growth factor receptor (EGFR) mutations in non-small cell lung cancer. Semin Cancer Biol.

[14] Schieppati A, Henter JI, Daina E, Aperia A (2008). Why rare diseases are an important medical and social issue. Lancet.

